# Prevalence and predictors of type 2 diabetes complications: a single centre observation

**DOI:** 10.4314/ahs.v23i3.37

**Published:** 2023-09

**Authors:** Chinonyerem O Iheanacho, Tolulope Folashade Akhumi, Uchenna I H Eze, Winifred A Ojieabu

**Affiliations:** 1 Department of Clinical Pharmacy and Public Health, Faculty of Pharmacy, University of Calabar, Calabar, Nigeria; 2 Department of Clinical Pharmacy and Biopharmacy, Faculty of Pharmacy, Olabisi Onabanjo University, Sagamu, Nigeria

**Keywords:** Complications, Nigeria, prevalence, socio-demographics predictors, type 2 diabetes

## Abstract

**Background:**

Diabetes complications are a major burden on persons living with diabetes and the health care systems.

**Objectives:**

The study assessed the glycemic control, prevalence and predictors of type 2 diabetes complications among patients in a healthcare centre.

**Methods:**

Two hundred adults who had type 2 diabetes in a general hospital were recruited for the study. Cross-sectional and retrospective surveys were used to determine prevalence, number and types of complications in the patients. SPSS version 21 was used for descriptive analysis and Chi-square (p<0.05).

**Results:**

A total of 200 (100%) respondents participated in the study and 97 (48.5%) had poor glycemic control. Mean number of complications per patient was 2.48 ± 1.22. Number of complications per person and type of complications were significantly associated with Age (p = 0.000 and p = 0.000, respectively), Gender (p = 0.008 and p = 0.031, respectively) and Occupation (p=0.000 and p=0.006, respectively). Marital status (p = 0.032) and years of diagnosis (p=0.021) were also associated with type of complications. The majority of patients 64 (32.0%) were admitted in the previous year for diabetes-related complications. Majority 159 (79.5%) had ≥ 2 number of complications from the observed 497 complications.

**Conclusions:**

Poor glycemic control and high prevalence of complications were observed. Also, socio-demographic characteristics were likely predictors of number and type of complications. These findings are essential for improved planning and prioritizing of diabetes care.

## Introduction

The rising incidence of type 2 diabetes has been observed over the years, and this is attributed to several factors which range between non-modifiable and modifiable factors 1. Type 2 diabetes involves persistent hyperglycemia resulting from insulin insufficiency or insulin resistance 2. Although high prevalence of diabetes has been observed in the African region, slight variations have been noted across the region. It has been reported to be higher in the urban areas than the rural areas in North Africa 3. In 2017, the prevalence of diabetes mellitus was reported as 4.3% in a suburban population in Nigeria 4, and 7.9 % in an urban city 5. Prevention of the risks of complications in type 2 diabetes is associated with adequate long term glycemic control which is a key determinant of the disease prognosis 6. Diabetes complications appear to be a great health threat to persons with diabetes, however individualised therapy and identification of relevant predictors are a reliable approach for effective prevention and specific treatment of these complications, especially in the high-risk individuals 7.

Significant morbidity and complications associated with diabetes constitute a major burden 8. Studies have shown high prevalence of diabetes complications 9,10, arising from inadequate self-care, insufficient education, and non-adherence to prescribed medicines, among others. More specifically, prevalence of diabetic retinopathy ranges between 8.1% and 41.5%, while neuropathy ranges between 21.9% and 60.0% in prevalence in Northern Africa 3. Similarly, a prevalence of diabetes complications ranging between 14% and 20%, and a mortality of 28.2% was reported in a recent study of a Nigerian population 11. Microvascular complications are noted to be more prevalent in persons with diabetes in the Nigerian population than macrovascular complications 12. Prolonged inadequate control of hyperglycemia is associated with, and may effectively predict these complications 9,11,13,14. Therefore, diabetes-related complications are a major challenge requiring both medical treatment and self-care for its prevention. The study assessed the glycemic values, prevalence and predictors of type 2 diabetes in adults attending a health facility.

## Methods

### Study setting

This study was conducted at the state government-owned general hospital in Ijebu Ode, located in Southwest Nigeria. The facility serves as a first point of call for patients, and a referral centre for primary health facilities around the town. The medical out-patients' clinic largely caters for persons living with diabetes and hypertension, and the hospital also provides referrals to tertiary facilities where need be.

### Study design and sampling method

Cross-sectional and retrospective (hospital records) survey were conducted. Patients with type 2 diabetes were consecutively recruited over a period of 3 months, and 200 diabetes patients who visited the hospital were included in the study. Sample size was based on estimated monthly average patient turnout at the hospital. From a monthly average of 112 diabetes patients, a sample size of 189 was calculated, including a 5% attrition, and a round figure of 200 patients was recruited for the study.

### Study population

All adult diabetes patients who visited the hospital formed the study population. The study included adult in-patients and out-patients of 30 years old and above, who had type 2 diabetes, and visited the endocrinology department of the healthcare facility. Persons from whom informed consent was not obtained were excluded from the study.

### Study instrument

Diabetes complications are a preventable outcome of diabetes, and its prevalence have remained a major concern among care givers. The questionnaire was hence, composed of questions that assessed the diabetes-related medical history and prevalence of diabetes in the patients. A structured self-completion questionnaire was administered to the study participants and its constructs measured the prevalence of diabetes-related complications and diabetes-related medical history of type 2 diabetes patients. The questionnaire consisted of 2 sections, section A elicited socio-demographic data of the respondents, and Section B obtained data on the respondents' medical history, current drug treatment and diabetes complications. The questionnaire was validated by expert assessment (3 endocrinologists) and pretested among 20 persons of similar demographic characteristics, after which the questions were revised to exclude ambiguity.

### Data collection and study outcome measures

Data were collected consecutively on each clinic day, over a 3-month period, using a self-completion questionnaire. Care givers of patients were also interviewed to confirm the responses provided by the patients. Patients' records were kept to avoid duplicate enrollment of patients. Information on diabetes-related complications were obtained from the patients and patients' records.

Retrospective survey (hospital records) was used to validate the patients' responses. The respondents' case files were accessed and information on drug therapy and complications due to diabetes were obtained for each patient. Areas of ambiguity on the occurrence of diabetes complications in the patients' medical charts were confirmed from their physicians during the investigation.

The main study outcome measures were prevalence of diabetes complications and associations of socio-demographics with number and type of diabetes complications. The secondary outcome measure was the patients' glycemic values.

### Data analyses

Data entry and analysis were conducted using IBM Statistical Package for Social Sciences (SPSS) version 21, and descriptive statistics was done. Categorical variables were described by frequencies and percentages, and presented in Tables and Figures.

Analysis of complications was based on number of complications in the patients. This was classified into 3 for easy analysis as: a) no complications, b) one complication, and c) two or more complications. Based on type of complications, patients were classified as having: a) no complications b) macro vascular complications c) micro-vascular complications d) both macro and micro vascular complications

Chi square test was used for further analysis to test for the relationship between socio-demographic variables and number/type of complication. P<0.05 was considered statistically significant.

### Ethical considerations

Ethical approval with reference number: IT/1/VOLI, was obtained from the state hospital prior to the study. Informed consent was also obtained from the patients, and confidentiality was ensured during and after the study.

## Results

A total of 200 respondents participated in the study and complete responses were obtained, giving a response rate of 100 %. The mean blood glucose value was 142.12 ± 65.31 mg/dl, while the mean number of complications per patient was 2.48 ± 1.22.

Majority of the respondents were 60 years old and above; 141 (70.5%), females; 138 (68.0%), married; 120 (60.0%), and had low educational background with over a half; 108 (54.0 %) having less than secondary education. Age (p < 0.001), gender (p = 0.008) and occupation (p < 0.001) were significantly associated with number of complications in the respondents. See Table 1.

**Table 1:** Association of socio-demographic characteristics and number of diabetes complications among type 2 diabetes patients

Variables	Frequency (n = 200)	Percentage (%)	Number of Complications			Significance
			None	One	≥ Two	
**Age (years)**						
30 – 39	5	2.5	2 (1.0%)	2 (1.0%)	1(0.5%)	X^2^ = 34.580;
40 – 49	14	7.0	0 (0.0%)	7 (3.5%)	7 (3.5%)	df = 6, p =
50 – 59	40	20.0	2 (1.0%)	7 (3.5%)	31 (15.5%)	<0.001*
≥60	141	70.5	4 (2.0%)	17 (8.5%)	120(60.0%)	
**Gender**						X^2^ = 9.730; df
Male	64	32	6 (3.0%)	6 (3.0%)	52 (26.0%)	= 2; p
Female	136	68	2 (1.0)	27 (13.5%)	107(53.5%)	= 0.008*
**Marital status**						
Single	2	1.0	0 (0.0%)	1 (0.5%)	1 (0.5%)	X^2^ = 7.834; df
Married	120	60.0	7 (3.5%)	19 (9.5%)	94(47.0%)	= 6; p = 0.25
Separated	5	2.5	2 (1.0%)	1 (0.5%)	2 (1.0%)	
Divorced	4	2.0	2 (1.0%)	1 (0.5%)	1 (0.5%)	
Widowed	70	35.0	0 (0.0%)	11 (5.5)	59 (29.5%)	
**Education**						
No formal education	51	25.5	0 (0.0%)	9 (4.5%)	42(21.0%)	X^2^ = 7.682; df = 6; p = 0.262
Primary	57	28.5	1 (0.5%)	8 (4.0%)	48 (24.0%)	
Secondary	49	24.5	3 (1.5%)	10 (5.0%)	36 (18.0%)	
Tertiary	43	21.5	4 (2.0%)	6 (3.0%)	33 (16.5%)	
**Income Naira**						
No steady income	133	66.5	1 (0.5%)	5 (2.5%)	24 (12.0%)	X^2^ = 7.787; df = 6; p = 0.254
<50, 000	30	15.0	2 (1.0%)	5 (2.5%)	18 (9.0%)	
50 – 125, 000	25	12.5	2 (1.0%)	1 (0.5%)	9 (4.5%)	
>125, 000	12	6.0	3 (1.5%)	22 (11.0%)	108(54.0%)	
**Occupation**						
Self-employed	63	31.5	0 (0.0%)	15 (7.5%)	48 (24.0%)	X^2^ = 30.192;
Employee/Civil servant	13	6.5	3 (1.5%)	5 (2.5%)	5 (2.5%)	df = 8 p = <0.001*
Retiree	41	20.5	3 (1.5%)	2 (1.0%)	36 (18.0%)	
Vocational	14	7.0	1 (0.5%)	2 (1.0%)	11 (5.5%)	
Unemployed	69	34.5	1 (0.5%)	9 (4.5%)	59 (29.5)	

* Statistical significant; One US dollar = 450 Naira

Table 2 shows the patients' diabetes-related medical history where it is seen that most 145 (72.5%) patients reported non-familial history of diabetes mellitus. Diabetes complications was observed in 192 (96.0%) of study participants, with a large number having both microvascular and macrovascular complications 133 (66.5%). Majority also had two or more complications 159 (79.5%). Overall, the majority 152 (76.0%) did not monitor their blood glucose at home, and about one third 72 (36.0 %) had hospital admission in the last one year. Of the number admitted in the past one year, majority 64 (32.0%) were for diabetes-related complications. Also, a large number 186 (93.0%) took oral anti-diabetic drugs and almost half 97 (48.5%) had fasting glycemic values of above 126 mg/ dl. Over half of the patients 108 (54.0%) had their follow-up clinic visits monthly and less than half had it fortnightly. Meanwhile only very few had it weekly 4 (2.0%).

**Table 2:** Diabetes-related medical history of respondents with type 2 diabetes in a Nigerian Health facility

Variable	Frequency (n)	Percentage (%)
**Family history of diabetes**		
Yes	55	27.5
No	145	72 .5
**Do you monitor blood sugar at home?**		
Yes	48	24
No	152	76
**Have you been admitted in the hospital for the past one year?**		
Yes	72	36
No	128	64
**If yes;**		
Diabetes-related complications	64	32
Other medical conditions	8	4
**Medications for diabetes**		
Pills	186	93
Pills and insulin	13	6.5
Only Insulin	1	0.5
**Glycemic value**		
>126mg/dl	97	48.5
≤126mg/dl	103	51 .5
**Number of complications**		
0	8	4.0
1	33	16.5
≥ 2	159	79 .5
**Type of complication**		
None	8	4.0
Microvascular	41	20.5
Macrovascular	18	9
Both	133	66 .5
**Follow-up**		
Monthly	108	54.0
Every 2 weeks	88	44.0
Weekly	4	2.0

**Figure 1:**
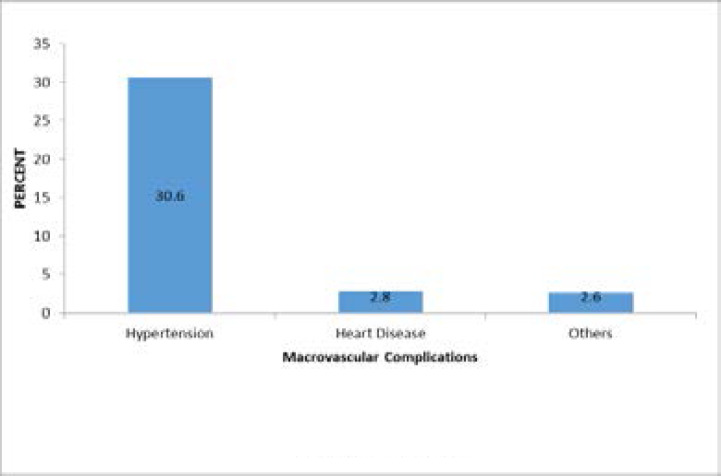
Occurrence of Types of Macrovascular Complications

**Figure 2:**
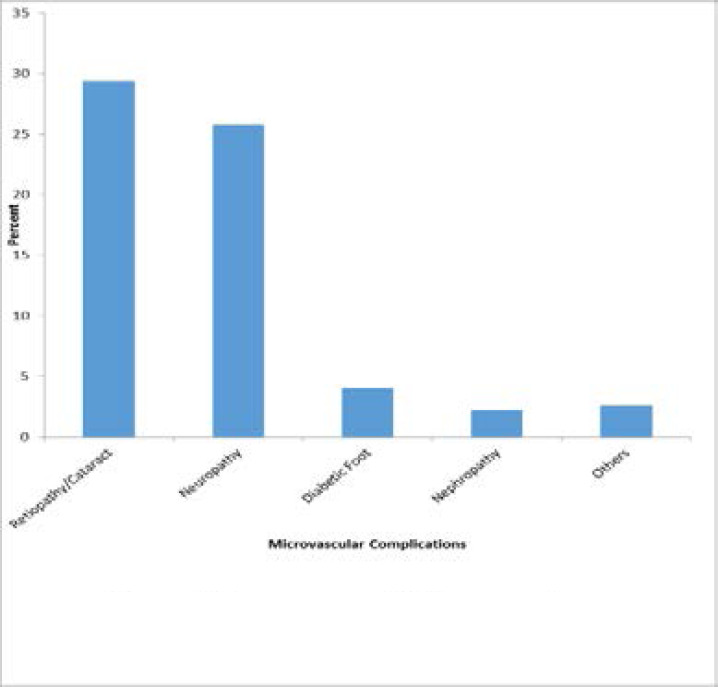
Occurrence of Microvascular Complications

A total of 497 complications were recorded among the 200 respondents, with microvascular complications accounting for 318(63.8%), while 179(36.02%) were macrovascular. Most occurring complication were Hypertension 152(30.6%), Retinopathy/Cataract 146 (29.4%) and neuropathy 128 (25.8%). Figures 1 and 2 show the types of complications.

Category/type of diabetes complications was associated with patients' socio-demographic characteristics, and significant associations were observed for age (p = 0.001), gender (p = 0.031), marital status (p = 0.032), occupation (p = 0.006) and years of diagnosis (p=0.021). See Table 3.

**Table 3:** Category/Types of diabetes complications and its socio-demographic associated incidence among type 2 diabetes patients

Variables	None	Microvascular complications	Macrovascular complications	Both	Significance
Age					
30 – 39	0 (0.0%)	3 (1.5%)	0 (0.0%)	0 (0.0%)	X^2^=35.874;
40 – 49	0 (0.0%)	5 (2.5%)	4 (2.0%)	5 (2.5%)	df = 9; p
50 – 59	0 (0.0%)	8 (4.0%)	3 (1.5%)	27(13.5%)	< 0.001*
≥ 60	0 (0.0%)	25 (12.5%)	11 (10.5%)	101 (50.5%)	
**Gender**					X^2^=8.890;
Male	6 (3.5%)	12 (6.0%)	3 (1.5%)	43 (21.5%)	df=3;
Female	2 (1.0%)	29 (14.5%)	15 (7.5%)	90 (45.0%)	p=0.031*
**Marital status**					
Single	0 (0.0%)	2 (1.0%)	0 (0.0%)	0 (0.0%)	X^2^= 18.322;
Married	0 (0.0%)	21 (10.5%)	12 (6.0%)	80(40.0%)	df=9; **p =**
Separated/Divorced	0 (0.0%)	2 (1.0%)	2 (1.0%)	3 (1.5%)	**0.032**
Widowed	0 (0.0%)	16 (8.0%)	4 (2.0%)	50(25.0%)	
**Religion**					X^2^= 3.183; df
Christian	7 (3.5%)	25 (12.5%)	10 (5.0%)	75 (37.5%)	= 3; p =
Muslim	1 (0.5%)	16 (8.0%)	8 (4.0%)	58(29.0%)	0.364
**Educational Qualification**					
None	0 (0.0%)	11 (5.5%)	6 (3.0%)	34 (17.0%)	X^2^= 9.170; df
Primary	1 (0.5%)	10 (5.0%)	5 (2.5%)	41 (20.5%)	= 9; p =
Secondary	3 (1.5)	13 (6.5%)	4 (2.0%)	29 (14.5%)	0.442
Tertiary	4 (2.0)	7 (3.5%)	3 (1.5%)	29 (14.5%)	
**Occupation**					X^2^ = 27.593;
Self-employed	0 (0.0%)	17 (8.5%)	8 (4.0%)	38 (19.0%)	d=12; p
Employee/Civil servant	3 (1.5%)	4 (2.0%)	1 (0.5%)	5 (2.5%)	= 0.006*
Retiree	3 (1.5%)	3 (1.5%)	3 (3.5%)	32 (16.0%)	
Vocational	1 (0.5%)	4 (2.0%)	1 (0.5%)	8 (4.0%)	
None	1 (0.5%)	13 (6.5%)	5 (2.5%)	50(25.0%)	
**Income**					
Less	1 (0.5%)	6 (3.0%)	3 (1.5%)	20 (10.0%0	X^2^ = 10.873;
than ₦ 5,000					d= 9; p=
₦ 5, 000 - 20,000	2 (1.0%)	3 (1.5%)	4 (2.0%)	16 (8.0)	0.285
₦ 20,000 - 35,000	2 (1.0%)	1 (0.5%)	1 (0.5%)	8 (4.0%)	
₦ 35, 000 - 50,000	3 (1.5%)	31 (15.5%)	10 (5.5%)	89 (44.5%)	
**Years of diagnosis**					
< 1 year	3 (1.5%)	11 (5.5%)	9 (4.5%)	14 (7.0%)	X^2^ =27.012; d
1 -5	1 (0.5%)	17 (8.5%)	5 (2.5%)	70 (35.0%)	=
6 – 10	3 (1.5%)	9 (4.5%)	2 (1.0%)	31 (15.5%)	15; **p=0.021**
11 – 15	1 (1.0%)	2 (1.0%)	1 (0.5%)	11 (5.5%)	
16 – 20	0 (0.0%)	0 (0.0%)	0 (0.0%)	3 (1.5%)	
>20 years	0 (0.0%)	2 (1.0%)	1 (0.5%)	4 (2.0%)	

* Statistical significance

## Discussion

The study observed high prevalence of diabetes complications among study participants, with more than one complication occurring in the majority. Both microvascular and macrovascular complications were also observed to occur in a large number of the patients. It also highlights that age, gender and occupation were associated with number and type of diabetes complications among patients. Marital status and years of diabetes were also observed to be associated with the type of complication in patients.

High prevalence of diabetes complications was seen in study participants. Increased burden of type 2 diabetes with several undiagnosed cases have been observed in Nigeria 17, and socio-demographics and environmental factors may be a predisposing factor to this. Most times, the onset of complications usually present the need for initial hospital visits and diabetes diagnosis among Nigerians. This trend has resulted in high burden and high prevalence of diabetes complications in the Country, as also noted in previous studies 12. Therefore, measures to improve early diagnosis, and improved care are essential for reduced burden of diabetes complications.

As found in the socio-demographic associated pattern of diabetes-related complications, age, occupation and gender appear to be associated with the incidence of micro-vascular and macrovascular complications, as well as the incidence of the combination of both. Socio-economic and socio-demographic factors are independent risk factors for diabetes outcomes 18. This is particularly essential in low- and middle-income countries such as Nigeria. Therefore, it is pertinent that health practitioners do not only focus on diabetes parameters of patients, but also on associated socio-demographic variables. This will enable provision of targeted care to patients.

Meanwhile high blood pressure, retinopathy/cataract and neuropathy respectively, were the most prevalent complications observed. This is consistent with findings from a previous study among a Nigerian population 19. Also, diabetes-related microvascular complications have been noted to be more prevalent than macrovascular complications in Nigeria. This strongly suggests a need for the use of oral anti-diabetic drugs which have propensity for addressing cardiovascular and other microvascular risk factors. Particularly among these drugs is the newer incretin-based medications such as glucagon-like peptide-1 (GLP-1) receptor agonists and dipeptidyl peptidase 4 inhibitors, which should be strongly encouraged in treatment guidelines 16. Previous studies have also reported high prevalence of type 2 diabetes-related complications 3,10,11,14, while noting early diagnosis and treatment as possible means of its prevention 10. Nephropathy was the second most prevalent complication after hypoglycaemia in a similar study 9, while nephropathy, retinopathy and neuropathy were the most prevalent in a Tanzanian study^14^. Similarities in findings may be associated with similarities in socio-demographic characteristics of the populations.

From the study, majority of the patients did not monitor their blood glucose at home, and over a half of them had been hospitalized in the past one year, mostly for diabetes-related complications. This finding was also reported in a previous study in Nigeria 19. Self-care is a major component of diabetes treatment and as such, requires regular practice. Diabetes education is a major way to equip patients with the requisite knowledge and skill for self-care which includes self-blood glucose monitoring. However, education has been reported to be poor in some locations where a good number of diabetes patients never attended their diabetes education sessions 20. Self-blood glucose monitoring encourages normal blood glucose range in patients and therefore reduces the risks of diabetes-related complications 21.

Meanwhile, almost half of the respondents in this study had high glycemic values, indicating a possible poor control of diabetes among them. A number of issues including poor adherence to prescribed medicines, may have influenced this finding. Poor glycemic control increases the risks of occurrence of diabetes complications among patients. Meanwhile, proper glycemic control is seen to reduce morbidity and mortality in patients with diabetes, but this may not always be achieved as shown in some studies 19,20,22,23,24 as a result of several factors. In previous studies, a substantial number of patients had poor glycemic control 19, and the prevalence was average in Colombia, and this was said to be associated with family functions and treatment type 23. Similarly, very high prevalence of poor glycemic control was noted in Bangladesh 24. Majority of the patients in an Ethiopian study also had poor glycemic control, and this was attributed to duration of treatment and low educational level, among others 20. High rate of poor glycemic control was also seen in a previous study, where it was not associated with the duration of treatment and type of medication used 22. Lack of self-monitoring of blood sugar is closely associated with poor glycemic control 21. Poor glycemic control is a major determinant of the incidence and prevalence of micro-vascular and macro-vascular diabetes complications 11,13.

Oral anti-diabetic medications were mostly taken by the respondents. Similarly, more than two third of a study population were reportedly on oral medications for diabetes in a similar study 22. Metformin and glibenclamide were also most prescribed in a previous study 21. Metformin is recommended for first line use in the management of type 2 diabetes, except in patients for whom it is contraindicated 20,25. Reduced risks of diabetes complications are associated with adequate long term glycemic control, which is a major determinant of the disease prognosis. This could be facilitated by regular patient follow-up and monitoring. Patients' follow-up appeared to be regular in this study, with majority mostly given monthly appointment visits for monitoring. Inadequate follow-up was reportedly associated with poor glycemic control in type 2 diabetes patients 24. Monitoring of patients with chronic diseases enables better prognosis of the disease condition as it allows for early secondary preventions.

The respondents were mostly of low socio-economic background with majority being married and unemployed. Socio-economic background has been widely reported as major determinant of diabetes risk 26 and outcome 27, as this may directly affect awareness and self-care for secondary prevention. A previous study found low awareness of diabetes and diabetes screening among non-diabetics 28. Most of the respondents in this study did not have family history of diabetes. Although family history of type 2 diabetes is a strong independent risk for developing the disease 29, other factors have been reported as predisposing factors to the disease, such as gut microbiome composition,^7^ anthropometry 26 and environmental polluters 30.

The study was hospital-based and included only persons who visited the hospital during the study period, therefore findings may not be generally representative. Also, data for glycated HbA1c was not available for a more detailed assessment of glycemic control, blood glucose values were rather used. Irrespective of these limitations, the study has shed light on occurrence of diabetes-related complications and the most prevalent complications among study participants.

## Conclusion

High prevalence of diabetes complications was found in the study participants, and socio-demographic characteristics were associated with number and type of complications. Age, gender and occupations are likely predictors of number and type of diabetes complications in a patient. In addition, marital status and years of disease diagnosis are also likely predictors of type of complications. A high proportion of the patients also had high glycemic values and blood glucose self-monitoring was not common among them. These findings are essential for improved planning and prioritizing of diabetes care.
